# Annual acknowledgement of reviewers

**DOI:** 10.1186/1472-6874-13-11

**Published:** 2013-03-22

**Authors:** Emily Crow

**Affiliations:** 1 BioMed Central, 236 Grays Inn Rd, London, WC1X 8HB, United Kingdom

## Abstract

**Contributing reviewers:**

The editors of *BMC Women’s Health* would like to thank all our reviewers who have contributed to the journal in Volume 12 (2012).

## 

Hatem Abu Hashim

Egypt

Annsofie Adolfsson

Sweden

Mustafa Afifi

Oman

Ajmal Agha

Pakistan

Halida Akhter

USA

Hesham Al-Inany

Egypt

Redhwan Al-Najjar

Malaysia

Richard Amenyah

Ghana

Tarek Amin

Saudi Arabia

Debra Anderson

Australia

Chia-Liang Ang

Singapore

Nick Axford

United Kingdom

Syed Iqbal Azam

Pakistan

Stella Babalola

USA

Maggi Banning

United Kingdom

Ana Paula Barbosa

Portugal

Andreja Barisin

Croatia

Robin Bell

Australia

Deborah Billings

USA

Sarah Blagden

United Kingdom

Vincent Boama

United Kingdom

Jeff Burgess

USA

Jennifer Butt

South Africa

Sally Chan

Singapore

Mei-Wei Chang

USA

Joan Chrisler

USA

Spring Chenoa Cooper Robbins

Australia

Josip Culig

Hungary

Eugenia Cutillas

Spain

Leonardo De Pascalis

Italy

Tobias De Villiers

South Africa

Haitze Janko De Vries

Netherlands

Thérèse Delvaux

Benin

Lynette Denny

South Africa

Henry Doctor

Nigeria

Xinqi Dong

USA

Abdel-Hady El-Gilany

Egypt

Nulufer Erbil

Turkey

Maijaliisa Erkkola

Finland

Birgitta Essén

Sweden

Farah Farzaneh

Iran

Adesegun Fatusi

Nigeria

Richard Fielding

Hong Kong

Alfonso Frigerio

Italy

Julia Gage

USA

Bishan Garg

India

Maria Luisa Gasparri

Italy

Brenda Geiger

Israel

Rebecca Giguere

USA

Paolo Giorgi Rossi

Italy

Elizabeth Greene

USA

Jhumka Gupta

USA

Arja Häggman-Laitila

Finland

Marcia Hall

United Kingdom

Keith Hansen

USA

Juanita Hatcher

Canada

Rohana Haththotuwa

Sri Lanka

Ruth Heisey

Canada

Jose Alberto Hernandez Bueno

Mexico

Peter Hillemanns

Germany

Myra Hunter

United Kingdom

Sara Husain

United Kingdom

Ephrat Huss

Israel

Lete Ignacio

Spain

Jennifer Jabson

USA

Paweł Jagodziński

Poland

Albrecht Jahn

Germany

Vesna Juresa

Croatia

Mairead Kiely

Ireland

Laurence Kirmayer

Canada

Tellervo Korhonen

Finland

Jeffrey Kromrey

USA

Patrick Lacor

Belgium

Toine Lagro-Janssen

Netherlands

Claas Lahmann

Germany

Maria Lahuerta

USA

Joohee Lee

USA

Deborah Legorreta

Mexico

Amy Levi

USA

Chih-Chia Liang

Taiwan

Harri Lindholm

Finland

Sherry Lipsky

USA

Simin Liu

USA

Bebe Loff

Australia

Riitta Luoto

Finland

Antonio Maccio

Italy

Maria Yolanda Makuch

Brazil

Jean Martial Mari

France

Ma.Luisa Marvan

Mexico

Webster Mavhu

Zimbabwe

Dimitrios Mavrelos

United Kingdom

Florian Mayr

USA

Hiten Mistry

United Kingdom

Aree Moon

South Korea

Steven Narod

Canada

Mzikazi Nduna

South Africa

Johanna Helena Nel

South Africa

Anta Nelson

USA

Gins Northington

USA

Uchenna Nwagha

Nigeria

Bright Nwaru

Finland

Samuel Obi

Nigeria

Adeola Olaitan

United Kingdom

Ken Ong

United Kingdom

Emanuel Orozco

Mexico

Kristina Orth-Gomer

Sweden

Yutaka Osuga

Japan

Mary Panjari

Australia

Electra Paskett

USA

Malika Patel

South Africa

Elizabeth Peel

United Kingdom

Debbie Penava

Canada

Patrick Petignat

Switzerland

Felice Petraglia

Italy

Anna Petrova

USA

Ken Phillips

USA

Bee Koon Poh

Malaysia

Antonio Rizzo

Italy

Saulo Vasconcelos Rocha

Brazil

Masoud Roudbari

Iran

Pagona Roussi

Greece

Heather Rowe

Australia

Aase Sagen

Norway

Khlood Salman

USA

Anna Sapino

Italy

Rebecca Say

United Kingdom

James Segars

USA

Julie Serovich

USA

Bettina Shell-Duncan

USA

Frances Shiely

Ireland

Alicja Sieminska

Poland

Jennifer Smit

South Africa

Robert Smith

USA

Razvan Socolov

Romania

Kate Soldan

United Kingdom

Marni Sommer

USA

Sandra Sosa-Rubi

Mexico

Petrus Steyn

South Africa

Jon Stone

United Kingdom

William Stones

Kenya

Maryam Tajvar

United Kingdom

Stephen Taplin

USA

Viju Thomas

South Africa

Gill Thomson

United Kingdom

Petra Thorn

Germany

Elena Toffol

Finland

Kirsti Uusi-Rasi

Finland

Zephne Van Der Spuy

South Africa

Solveig Vatnar

Norway

Ilze Viberga

Latvia

Elisabeth Vieira

Brazil

Ariel Weissman

Israel

Beverly Winikoff

USA

Li Ping Wong

Malaysia

Mingyan Xiang

United Kingdom

Wanghong Xu

China

Cheng-Har Yip

Malaysia

Pawel Zagozdzon

Poland

Manuel Zorzi

Italy

Fulvio Zullo

Italy

## Pre-publication history

The pre-publication history for this paper can be accessed here:

http://www.biomedcentral.com/1472-6874/13/11/prepub

